# Prediction of Solid-State Form of SLS 3D Printed Medicines Using NIR and Raman Spectroscopy

**DOI:** 10.3390/pharmaceutics14030589

**Published:** 2022-03-08

**Authors:** Sarah J. Trenfield, Patricija Januskaite, Alvaro Goyanes, David Wilsdon, Martin Rowland, Simon Gaisford, Abdul W. Basit

**Affiliations:** 1 UCL School of Pharmacy, University College London, 29-39 Brunswick Square, London WC1N 1AX, UK; sarah.trenfield.16@alumni.ucl.ac.uk (S.J.T.); patricija.januskaite.18@ucl.ac.uk (P.J.); a.goyanes@fabrx.co.uk (A.G.); s.gaisford@ucl.ac.uk (S.G.); 2 Departamento de Farmacología, Farmacia y Tecnología Farmacéutica, I + D Farma (GI-1645), Facultad de Farmacia, Universidade de Santiago de Compostela, 15782 Santiago de Compostela, Spain; 3 Worldwide Research and Development, Pfizer Inc., Groton, CT 06340, USA; david.wilsdon@pfizer.com; 4 Pfizer Ltd., Drug Product Design, Discovery Park, Ramsgate Road, Sandwich CT13 9ND, UK; martin.rowland@pfizer.com; 5 FabRx Ltd., Henwood House, Henwood, Ashford TN24 8DH, UK

**Keywords:** 3D printing, additive manufacturing, 3D printed drug products, manufacturing formulations, process analytical technology (PAT), oral drug delivery systems and technologies, printlets, personalized pharmaceuticals, digital healthcare

## Abstract

Selective laser sintering (SLS) 3D printing is capable of revolutionising pharmaceutical manufacturing, by producing amorphous solid dispersions in a one-step manufacturing process. Here, 3D-printed formulations loaded with a model BCS class II drug (20% *w*/*w* itraconazole) and three grades of hydroxypropyl cellulose (HPC) polymer (-SSL, -SL and -L) were produced using SLS 3D printing. Interestingly, the polymers with higher molecular weights (HPC-L and -SL) were found to undergo a uniform sintering process, attributed to the better powder flow characteristics, compared with the lower molecular weight grade (HPC-SSL). XRPD analyses found that the SLS 3D printing process resulted in amorphous conversion of itraconazole for all three polymers, with HPC-SSL retaining a small amount of crystallinity on the drug product surface. The use of process analytical technologies (PAT), including near infrared (NIR) and Raman spectroscopy, was evaluated, to predict the amorphous content, qualitatively and quantitatively, within itraconazole-loaded formulations. Calibration models were developed using partial least squares (PLS) regression, which successfully predicted amorphous content across the range of 0–20% *w*/*w*. The models demonstrated excellent linearity (R^2^ = 0.998 and 0.998) and accuracy (RMSEP = 1.04% and 0.63%) for NIR and Raman spectroscopy models, respectively. Overall, this article demonstrates the feasibility of SLS 3D printing to produce solid dispersions containing a BCS II drug, and the potential for NIR and Raman spectroscopy to quantify amorphous content as a non-destructive quality control measure at the point-of-care.

## 1. Introduction

Orally administered drug products are by far the preferred form of medication, recognised as being low cost, simple to administer, and having a high acceptability in patients [1]. However, around 40% of these products possess low water solubility and poor bioavailability, alongside a staggering 90% of new molecules, resulting in a high failure rate during drug development [2]. Due to these statistics, using enabling technologies to formulate drugs in the amorphous form as solid amorphous dispersions (SADs) has been of major interest in the pharmaceutical field in recent years. Indeed, amorphous materials lack long range structural order, meaning the main barrier to dissolution is reduced, enabling an improved water solubility and bioavailability profile [3].

Formation of SADs suitable for oral delivery (such as tablets or capsules) often requires several processing stages (e.g., hot melt extrusion (HME), spray-drying, or freeze-drying processes), which are laborious, costly, and time-consuming for production [4]. In recent years, three-dimensional (3D) printing (3DP) has been proven as an enabling technology and has been shown to be capable of creating SADs in a rapid, single-step, and dose-flexible process at the point-of-care [5,6,7,8,9,10,11,12,13,14]. A particularly promising 3DP technology in this area is selective laser sintering (SLS).

Using a layer-by-layer approach, SLS 3DP employs a diode laser, which is directed onto a drug-loaded powder-bed feedstock, resulting in partial or full sintering, to bind powder particles together [15,16,17]. Once the layer is sintered, a roller/blade distributes a fresh layer of powder on top of the sintered material, which is repeated to produce 3D-printed tablets (Printlets™). To date, SLS 3DP has been used to produce formulations containing flexible dosages, geometries, drug release profiles, and multi-drug combinations in a single-step process [18,19,20,21].

SLS 3DP has also shown great potential to be used as an enabling technology for low-solubility compounds, by producing solid dispersions during the printing process. Due to the combined application of heat and laser energy during the SLS process, an increased localised temperature first causes a sintering of the polymer and drug particles, followed by a rapid cooling to the surface temperature of the printer, which maintains the drug as intertwined in the polymer matrix in an amorphous form. Indeed, to date, a wide range of drugs, including paracetamol [22,23], ritonavir and copovidone [24], lopinavir [25], ibuprofen [26], indomethacin [27], ondansetron [28], and diclofenac sodium [29], as well as amlodipine and lisinopril [30], have been converted to a full or partial amorphous form post-printing using SLS technology.

Evaluating amorphous/crystalline content plays an important role in the pharmaceutical industry. To date, X-ray powder diffraction (XRPD) has been used as the ‘gold standard’ for analysing amorphous content [31]. However, the detection limit of this technique is generally low (~4% content), because of its sensitivity to the presence of crystallinity [32]. Differential scanning calorimetry (DSC) is another common technique, using the glass transition (Tg) as the assay parameter. However, it is a slow and destructive method, and there is often difficulty in measuring the Tg step height precisely. Whilst these techniques are widely used in pharmaceutical analysis, they are unsuitable for the real-time analysis of 3D-printed formulations, due to being relatively slow, requiring expensive equipment and trained personnel, and (in the case of DSC) being destructive. As such, alternative, non-destructive analytical techniques should be investigated for potential advantages over the standards used today. Spectroscopic methods, including near-infrared (NIR) spectroscopy [33] and Raman spectroscopy [34], have been extensively researched and have proven to be highly effective and accurate in solid state determination, and have been indicated as potential solutions to the aforementioned challenges [35,36].

The aim of this research was, for the first time, to compare the ability of NIR and Raman spectroscopy to analyse amorphous content qualitatively and quantitatively in SLS 3D-printed formulations composed of a model BCS class II drug (itraconazole), and three grades of hydroxypropyl cellulose (HPC) polymer (-SSL, -SL and -L). The physical characteristics of the formulation blends (particle shape, size, and powder flow), as well as printlet properties (hardness, and weight variation) were also assessed.

## 2. Materials and Methods

Itraconazole USP grade (Fagron, UK) (MW 705.64, water solubility: 1–4 μg/mL) was used as the model drug. Three different hydroxypropyl cellulose (HPC) grades were evaluated: HPC-SSL (MW 60,000), HPC-SL (MW 100,000), and HPC-L (MW 140,000) (Nippon Soda, Tokyo, Japan). Candurin^®^ Gold sheen was purchased from Merck, Poole, UK.

### 2.1. Amorphous form Production

Crystalline itraconazole powder was heated for 15 mins in a foil covered glass beaker on a hot plate, a couple of degrees above its melting point (itraconazole = 175 °C). Once turned molten, the foil was removed from the beaker and placed inside a mortar, into which liquid nitrogen was rapidly poured, to quench cool the molten drug. Once the liquid nitrogen had evaporated, the amorphous drug was gently ground into smaller particles using a pestle and mortar and transferred to a desiccator over silica gel, to reach room temperature before analysis.

### 2.2. Formation of Calibration Samples

The sieved polymer (180 μm sieve), 3% *w*/*w* Candurin^®^ Gold sheen and crystalline drug were mixed thoroughly in a pestle and mortar. Once mixed, the required quench cooled % amorphous drug was added and mixed carefully with a spatula, to not disrupt the amorphous solid state of the drug. Each individual calibration sample composition is displayed below in Table 1. Calibration samples were presented to the NIR and Raman spectrometer either in a pure powdered formulation blend or compressed into squares (20 mm × 20 mm) using a manual hydraulic press at a force of 5 PSI (Specac Ltd., Orpington, UK).

### 2.3. Printing Process

For each formulation, 15 g of powder mixture was made by mixing 20% *w*/*w* of the drug and 77% *w*/*w* of the sieved polymer (through a 180 μm sieve) in a pestle and mortar (Table 1). Then, 3% *w*/*w* of the colourant Candurin^®^ Gold Sheen was added to each formulation as an absorbent, to enhance laser energy absorption and ensure printability. Part of the powder was then transferred to a SLS printer (Sintratec Kit, AG, Brugg, Switzerland) to formulate the printlets and discs. Subsequently, 123D Design was used to design the shape of the cylindrical discs (23 mm diameter × 1 mm height) and printlets (10 mm diameter × 3.6 mm height). The 3D models were then exported as a stereolithography (.stl) file into the 3D printer Sintratec central software.

Part of the powder mixture was added to the building platform (130 × 130 × 30 mm), which was set in its highest position, where the blade was moved across to flatten and create an even and homogenous powder bed for printing. The printing surface temperature was set at 100 °C, the chamber temperature at 80 °C, and the laser scanning speed at 300 mm/s. The printing process began with the activation of a 2.3 W blue diode laser (445 nm), to sinter the first layer of powder onto the building platform, based on the pattern in the .stl file. As soon as the laser had stopped sintering the first layer, the roller distributed a new powder layer over the previously sintered area. This process was repeated layer-by-layer, until the desired object was completed. Individual discs/printlets were removed from the printer once cooled, and any excess unsintered powder was brushed off.

### 2.4. XRPD Analysis

Drug, polymer, and excipient mixtures were 3D printed into 23 mm diameter × 1 mm height discs for analysis. Samples of pure drug and HPC polymers were also analysed. The XRPD patterns were collected in a Rigaku MiniFlex 600 (Rigaku, The Woodlands, TX, USA) using a Cu Kα X-ray source (λ = 1.5418 Å). The intensity and voltage applied were 15 mA and 40 kV. The angular range of data acquisition was 3–40° 2θ, with a stepwise size of 0.02° and a speed of 2°/min.

### 2.5. Near-Infrared Spectroscopy

NIR reflectance spectra were measured using a Fourier transform (FT)-NIR spectrometer (MPA, Bruker, Germany). Spectra were collected using the microsample setting (3-mm spot size) across wavenumbers of 12,500 to 3600 cm^−1^ and at a resolution of 8 cm^−1^, totalling 64 scans, which were averaged. Each disc was analysed at three different points, to avoid potential sampling errors and to reduce the variability caused by different surface effects. Powder calibration samples were scanned at three different points. The final spectrum for each sample was the average of the spectra recorded at the three positions for discs and three positions for powders. Collection of the data was performed using OPUS Version 6.5 software (Bruker, Billerica, MA, USA).

### 2.6. Raman Spectroscopy

The calibration samples, printed discs, unsintered formulation blends, and pure ingredients were evaluated using a RamanRXN Systems Raman Spectrometer (Kaiser Optical Systems Inc., Ann Arbor, MI, USA) equipped with a 400 mW 785 nm HPNIR Renishaw laser at 100% laser power. Samples were scanned using a connected PhAT non-contact optical probe (6 mm spot size, 250 mm focal length; Kaiser Optical Systems Inc., Ann Arbor, MI, USA). Spectra were collected over the range of 100–2000 cm ^−1^, with a 16-s exposure time and 4 accumulations. The final spectrum for each sample was the average of the spectra recorded at a minimum of three separate positions.

### 2.7. Multivariate Data Analysis

Calibration samples ranging from Cal 1 (20% *w*/*w* crystalline) to Cal 6 (0% *w*/*w* crystalline) were selected for calibration model development (*n* = 14), and four calibration samples were randomly selected for internal validation (*n* = 4). Multivariate data analysis was performed using MATLAB software version R2019a (The MathWorks, Torrance, CA, USA) with the PLS_Toolbox version 8.6 (Eigenvector, Wenatchee, WA, USA) for data pre-processing and modelling. Partial least squares (PLS) regression was performed on the datasets, to build calibration models. The models were internally cross-validated using leave-one-out cross-validation. Validation of the calibration models was performed according to guidance from the International Conference on Harmonization (ICH) Q2(R1) [37], European Medicines Agency (EMA) [38] and the Food and Drug Administration (FDA) [39], by assessing model specificity (expressed as the 1st latent variable; LV1), linearity (expressed as correlation coefficient, R^2^), and accuracy (expressed as the root mean square error of prediction; RMSEP).

### 2.8. Powder Flow Characteristics

#### 2.8.1. Bulk Density

Bulk density was determined by pouring pure HPC polymers (HPC-L, -SL, -SSL, and -UL) and itraconazole formulation blends into a 10-mL volumetric cylinder. The bulk density (*n* = 3) was calculated using Equation (1):

(1) ρb=M/V

where ρb = bulk density, *M* = mass of powder (g), *V* = volume of powder (mL).

#### 2.8.2. Tapped Density

An accurately weighed quantity of powder was carefully transferred into a 10-mL measuring cylinder. The cylinder was then tapped onto a wooden surface from the height of 2.5 cm, at one second intervals. The tapping was continued until no further change in volume (until a constant volume) was obtained (V_f_). The tapped density (*n* = 3) was calculated by Equation (2):

(2) ρt=M/V

where ρt = tapped density, *M* = mass of powder (g), *V* = volume of powder (mL).

#### 2.8.3. Compressibility Index and Hausner Ratio

The compressibility index and Hausner ratio were determined by measuring both the bulk density and tapped density of the powders. The compressibility index (Equation (3)) and the Hausner ratio (Equation (4)) were calculated as follows:

(3) Compressibility Index=100×(ρt−ρb)/ρb

(4) Hausner Ratio=ρt/ρb

### 2.9. Physical Properties of Printlets

The crushing strength of three printlets for each speed was measured using a traditional tablet hardness tester TBH 200 (Erweka GmbH, Heusenstamm, Germany), whereby an increasing force was applied perpendicularly to the printlet axis to opposite sides of a printlet until it fractured. The mean and standard deviation were calculated.

### 2.10. Determination of Drug Content

A single printlet (approximately 80 mg) of each HPC polymer formulation was placed into a volumetric flask with 1:1 ethanol:acetonitrile mixture (100 mL) under stirring until complete dissolution (*n* = 2). Solution samples were then filtered using a 0.22-μm filter (Millipore Ltd., Carrigtwohill, Ireland), and the drug concentration was determined using high-performance liquid chromatography (HPLC) (Hewlett-Packard 1050 Series HPLC system, Agilent Technologies, Cheadle, UK).

The validated HPLC assay, based on a previous study with itraconazole [11], involved the injection of 10-μL solution samples through a mobile phase, made up of an isocratic system containing 70% acetonitrile and 30% water, pumped at a flow rate of 1 mL/min. The HPLC analysis was run through an Eclipse Plus C18 column, 4.6 × 150 mm (Zorbax, Agilent technologies, Cheshire, UK), which was kept at 40 °C. The eluent was screened at a 260-nm wavelength and the itraconazole retention time was found to be approximately 5 min.

## 3. Results and Discussion

### 3.1. Development of 3D Printed Formulations

**Figure 1:**
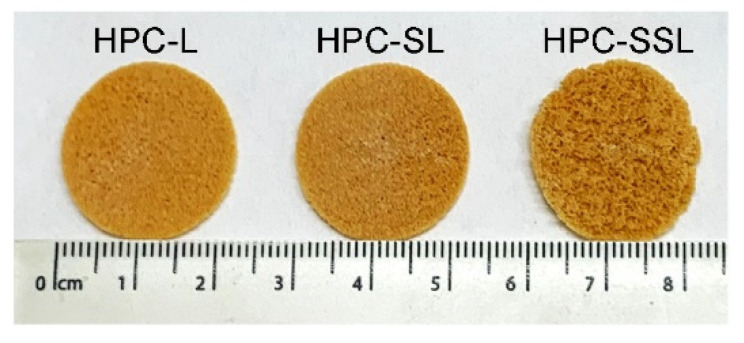
Images of itraconazole 3D printed discs using three different HPC grades (HPC-L, -SL, and -SSL, from left to right).

SLS 3DP was used to produce 20% *w*/*w* itraconazole discs (23 mm diameter × 1 mm height) (Figure 1) and printlets (10 mm diameter × 3.6 mm height) with three different HPC polymer grades (HPC-L, -SL, and -SSL). For formulation production, the SLS printing parameters were kept the same (100 °C surface temp, 80 °C chamber temp, 300 mm/s laser speed). Interestingly, the discs and printlets produced with the higher polymer molecular weights (HPC-L and HPC-SL) displayed a more uniform sintering on the formulation surface for both discs and printlets. Conversely, the lower molecular weight polymer (HPC-SSL) produced discs of unhomogenous surface morphology, which was hypothesised to be attributed to the poorer powder flow of the polymer.

To understand this phenomenon further, the physical characteristics of the printlets were evaluated. HPC-L and HPC-SL tablets had a higher weight and more desirable physical properties (i.e., increased breaking force and weight) compared with HPC-SSL formulations (Table 2). For all the printlets, the drug loading of itraconazole was within the British Pharmacopoeia (BP) requirements (ranging from 96.7–101.9%; Table 2).

It has been previously demonstrated that the degree of uniform sintering is highly dependent upon the particle size and morphology of the materials used [40]. Previous studies and HPC polymer data sheets have highlighted that higher MW HPC polymers have larger median particle sizes (160 μm for both HPC-L and HPC-SL) compared with lower MW grade (85 μm for HPC-SSL) [41]. For SLS 3DP, powder particle size can markedly impact the degree of laser sintering and, hence, the binding of the particles. Interestingly, small, and irregularly shaped particles may contribute towards poor powder flow, due to the agglomeration of particles via Van der Waals forces, thereby forming craters and defects on the surface of the powder during layer distribution, and they may not be as easily deposited on the building platform, due to their poor flowability [17,42,43]. As such, the physical differences between the 3D-printed formulations may be attributed to the variation in particle size and morphology of the HPC polymer grades.

To further understand the impact of polymer grade on the physical characteristics of the 3D-printed formulations, bulk and tapped density measurements were carried out. The results showed that the pure HPC-L and HPC-SL polymers demonstrated ‘passable’ powder flow properties, indicated by a compressibility index between 21–25% and Hausner ratio between 1.26–1.34 (Table 3). HPC-SSL demonstrated ‘poor’ powder flow properties, indicated by a compressibility index between 26–31% and Hausner ratio between 1.35–1.45 (Table 3). Upon addition of 20% *w*/*w* itraconazole, the powder flow properties worsened for all polymers (i.e., compressibility index and Hausner ratio increased), likely due to the poor flow characteristics exhibited by itraconazole in its pure form. These findings highlight a critical need for adequate powder flow to produce drug products using the SLS printing process; and in the future, the compressibility index and Hausner ratio should be used as a basis to confirm the powder sintering effectiveness. To improve formulations with poor powder flow characteristics, the addition of lubricants and/or glidants could also be utilised.

**Figure 2:**
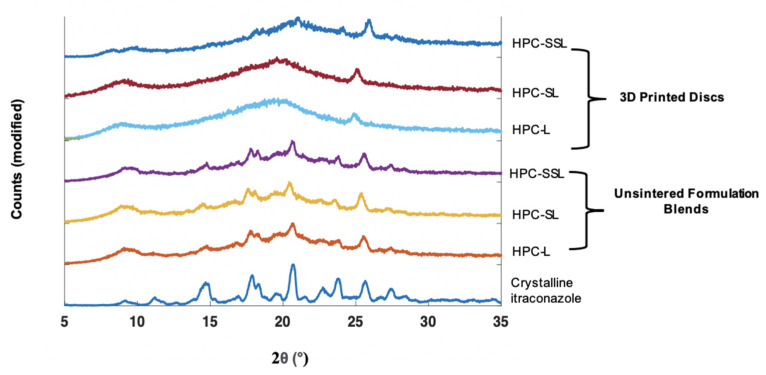
XRPD diffractograms of 20% *w*/*w* itraconazole-loaded 3D printed discs vs. powders.

XRPD analysis was performed to observe if the polymer grade had an influence on the drug formation in the 3D printed discs. In the unsintered powders, characteristic diffraction peaks relating to crystalline itraconazole can be seen at 17.5°, 20.8°, and 24° 2θ (Figure 2). In general, the SLS 3DP process was found to convert itraconazole from the crystalline to the amorphous form, indicated by the absence of diffraction peaks at the aforementioned angles; consistent with previous literature evaluating the use of direct powder extrusion (DPE) 3DP to formulate itraconazole-loaded formulations [11]. It was noted that 3D printed discs composed of HPC-SSL exhibited small diffraction peaks at 17.5° and 20.8°, indicating that itraconazole was retained in a partially crystalline form, which may have been due to unsintered powder material being retained on the printlet surface or pores, as found elsewhere [23,44,45].

### 3.2. Development of Calibration Samples

To create an assay for quantifying amorphous content, calibration samples with known quantities of each solid state were prepared. The amorphous form of itraconazole was produced by melting the crystalline form and, subsequently, using a quench cool method using liquid nitrogen. To evaluate whether the quench-cooled form was fully amorphous, XRPD, DSC, NIR, and Raman spectroscopy were used to confirm their physical solid state.

**Figure 3:**
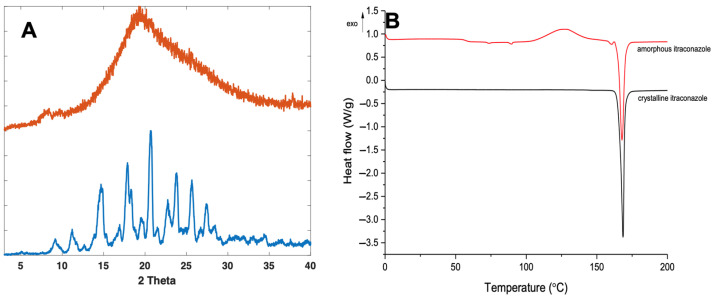
XRPD diffractogram (**A**) and DSC thermograph (**B**) of crystalline and quench-cooled (amorphous) itraconazole.

Crystalline itraconazole displayed characteristic XRPD diffraction peaks at 14.7°, 17.5°, 20.8°, 24°, 25.8°, and 27.5° 2θ (Figure 3A), consistent with form 1 as reported elsewhere [46,47]. Formation of amorphous itraconazole post quench cooling was confirmed by the presence of a broad halo in the XRPD diffractogram, with no characteristic Bragg’s diffraction peaks corresponding with the crystalline form (Figure 3A). 

For further confirmation, DSC analysis was performed on both the amorphous and crystalline forms of itraconazole. For the crystalline form, a characteristic melting endotherm at around 170 °C was observed (Figure 3B), which was demonstrated in other studies [46,47,48]. The quench-cooled sample was confirmed as amorphous, as shown by the change in baseline, representing the Tg, at ~58 °C for itraconazole [49,50]. However, a characteristic melting endotherm at 170 °C was also observed for the amorphous form. It is well known that quench-cooled amorphous compounds can be prone to recrystallisation inside the DSC, due to elevated temperatures, enabling the free movement and re-ordering of molecules into a regular crystalline arrangement [51]. As such, a recrystallisation exotherm can be observed at 130 °C, followed by a melting endotherm at 170 °C of the now partially crystalline form of itraconazole, a phenomenon which has been reported elsewhere [52].

**Figure 4:**
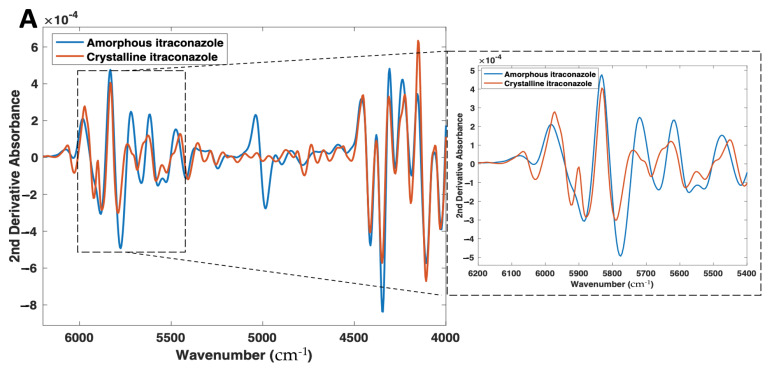

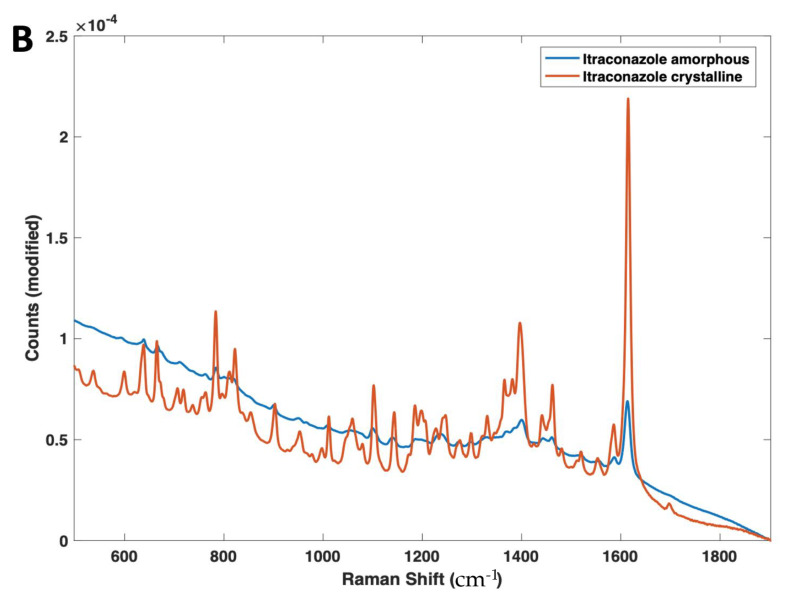
(**A**) 2nd derivative NIR spectra (filter width 21, 2nd polynomial) and (**B**) Raman spectra of both amorphous and crystalline itraconazole forms.

Differences in the NIR and Raman spectra were observed between the crystalline and amorphous forms (Figure 4A,B). With NIR spectroscopy, spectral changes, due to the solid state could be seen throughout the 4000–6200 cm^−1^ wavenumber range (Figure 4A). Most notably, the peaks at 5800–5900 cm^−1^, which represent the first overtone vibrations of the -C-H functional groups [53], exhibit a distinct morphology for the crystalline form (double peak), compared with its amorphous form (single broad peak). At the peak located at 5800 cm^−1^ for the crystalline material, an increase in peak intensity, as well as a peak shift to 5775 cm^−1^, is observed for the amorphous form. The peak at ~5000 cm^−1^ for amorphous itraconazole is attributed to the presence of water [54], likely due to its higher hygroscopicity compared with the crystalline form [55] and, as such, the region 4800–5200 cm^−1^ was excluded from the subsequent multivariate analysis.

These phenomena can be explained by a fundamental understanding of NIR spectroscopy, the spectra present distinct information on molecular structure and conformation from vibrations of atoms. NIR spectroscopy works on the principle that different solid state forms differ in the intermolecular hydrogen bonding pattern in a crystal lattice, which results in a shift or differing morphologies of absorption bands in NIR spectra. Previous studies have highlighted this occurrence for drugs including warfarin [56], sulfathiazole [57], and indomethacin [58], as well as excipients including lactose [59] and glycine [60].

Raman spectroscopy also found spectral differences based on the drug solid state (Figure 4B), with the amorphous form showing a marked reduction in peak heights across the entire Raman shift regions of 600–1800 cm^−1^, as well as a broadening of peaks, due to the lack of a long-range molecular order compared with the crystalline form; a trend that is commonly characteristic of amorphous materials [61]. In particular, the peak at 1618 cm^−1^, which represents the ν(C=C) functional group [62], displayed a significant reduction in intensity from the pure crystalline to the amorphous forms. Despite the same chemical composition, the Raman spectra of different solid state forms of the same drug can differ due to the presence of spatial order and long-range translational symmetry for crystalline solids compared with their amorphous counterparts [63].

In addition, the amorphous sample demonstrated a higher degree of fluorescence (shown by the increase in baseline at the lower Raman shifts). This is likely due to the prepared amorphous itraconazole having a yellow colour, a trait which is known to cause increased fluorescence and interference with peak heights [64].

### 3.3. Development of Calibration Models

**Figure 5:**
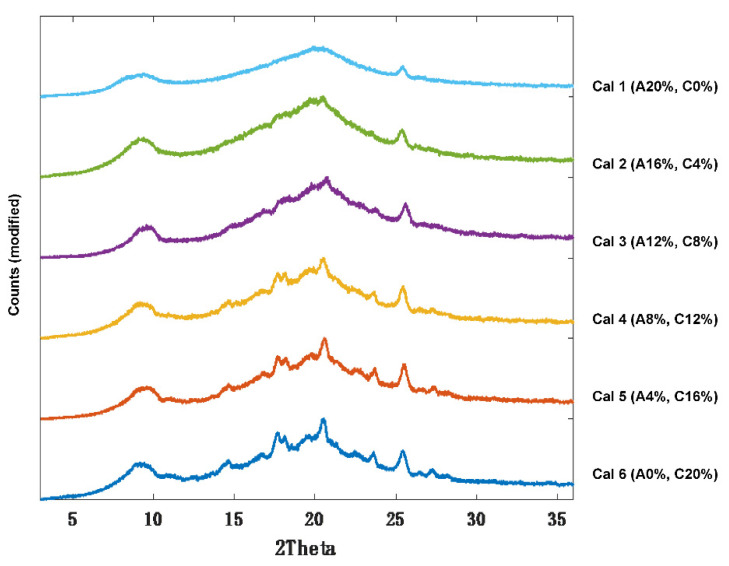
XRPD diffractograms of itraconazole calibration samples 1–6 (Cal 1–6).

Following successful preparation of amorphous itraconazole, calibration samples were prepared (Cal 1–6) by doping the HPC-L polymer with different ratios of crystalline:amorphous drug content; Cal 1 had 100:0 and Cal 6 0:100 (Table 1). As expected, XRPD analysis showed an increase in itraconazole diffraction peak intensities (in the region between 13–24° 2θ) as a function of crystalline drug content. As an example, Cal 6 (highest crystalline content) exhibited sharp and well resolved diffraction peaks, whereas Cal 1 (lowest crystalline content) demonstrated an absence of diffraction peaks (broad halo) (Figure 5). For Cals 1–5, the diffraction peak at 20.5° was detectable even at the lowest crystalline content (4% *w*/*w*).

Whilst XRPD can detect crystallinity of dosage forms, it is an inherently bulky, laborious, and expensive process, which would not be suitable for real-time analysis of 3D printed medicines and has poor sensitivity (>4% *w*/*w* crystallinity required). Alternative process analytical technologies (PAT) such as NIR and Raman spectroscopy have shown capabilities in quantifying crystalline and polymorph content [65]. As such, both NIR and Raman spectroscopic techniques were also evaluated for their ability to analyse amorphous content qualitatively and quantitatively in the developed 3D-printed formulations.

**Figure 6:**
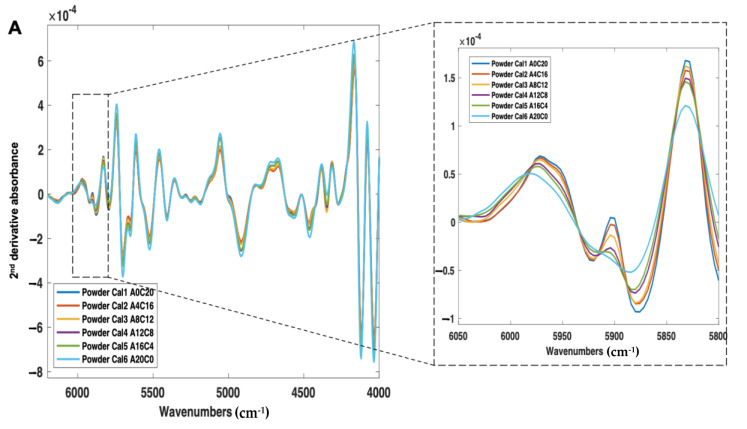

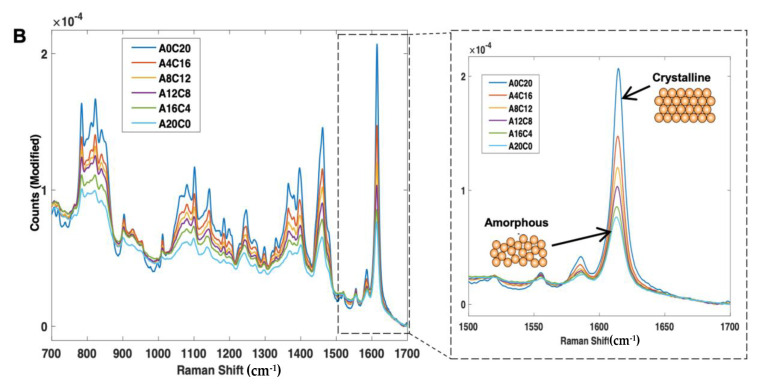
(**A**) 2nd derivative NIR spectra (filter width of 21, 2nd polynomial) and (**B**) Raman spectra (baseline corrected) of itraconazole calibration samples (Cal 1-6), ranging from 0% to 20% *w*/*w* crystalline content.

First, the calibration samples (Cal 1–6) were scanned using NIR and Raman spectroscopy (Figure 6A,B). With NIR spectroscopy, a change in peak morphology and intensity occurred as a function of crystalline content, even in the presence of a high polymer concentration (77% *w*/*w*) (Figure 6A). With Raman spectroscopy, consistent with the amorphous and crystalline spectra, as the sample amorphous content increased, the peak intensities across 700–1700 cm^−1^ were found to decrease, and the peak morphologies broadened (Figure 6B).

**Figure 7:**
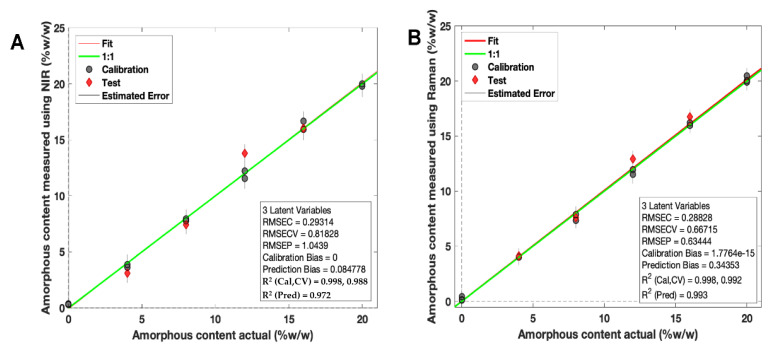
PLS regression calibration models based on (**A**) NIR spectroscopy and (**B**) Raman spectroscopy for amorphous itraconazole prediction. Grey points = calibration samples; red points = excluded test samples.

Two calibration models were developed using PLS regression for calibration samples scanned using both NIR and Raman spectroscopy (Figure 7A,B).

For NIR spectroscopy, the PLS model covered 4000–6200 cm^−1^ (water peak excluded) with a second derivative (Savitzky Golay method: filter width of 21 with a second polynomial [66], followed by standard normal variate (SNV) and mean-centering pre-processing techniques). The model developed had a high linearity (R^2^ Cal = 0.998) and accuracy, indicated by the root mean square error of prediction (RMSEP) value of 1.04% (Figure 7A). These values confirmed that the NIR test results were proportional to % amorphous content in the stated range (0–20% *w*/*w*). The specificity was high for solid state changes, indicated by an LV1 value of 90.03%.

For Raman spectroscopy, the PLS regression model was based on a previously reported method for itraconazole crystallinity quantification [67] (Figure 7B). First, the regions between 1000 and 1700 cm^−1^ were selected, followed by a Whittaker baseline correction (smoothness factor λ = 100,000 and *p* = asymmetry factor 0.001) and a normalisation to the area of peak 1614 cm^−1^ and mean-centering pre-processing method were used. The developed PLS model had a high linearity (R^2^ = 0.998) and an accuracy of 0.63%, indicated by the RMSEP value (Figure 7B). The specificity was high for solid state changes, indicated by an LV1 value of 86.31%.

It is well known that physical characteristics (such as tablet geometry, particle size, and compaction pressure) are sources of variability that can affect NIR spectra and, hence, model predictions [68]. As the calibration models here were developed using doped powdered samples, it was important to evaluate the applicability of the model to the 3D-printed discs (which had a different surface morphology to the calibration samples).

**Figure 8:**
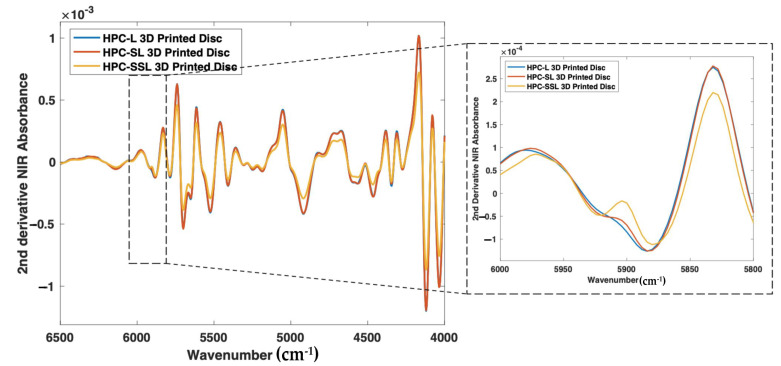
2nd derivative NIR spectra (filter width of 21, 2nd polynomial) of itraconazole-loaded 3D printed discs.

**Figure 9:**
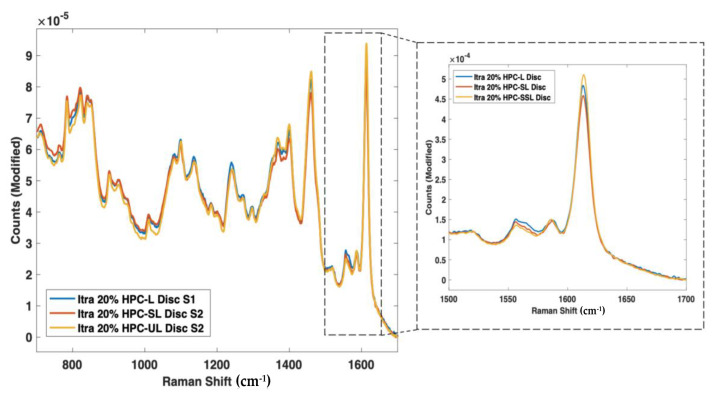
Raman spectra of itraconazole unsintered powders vs. 3D printed discs for (**blue line**) HPC-L, (**orange line**) HPC-SL, and (**yellow line**) HPC-SSL, focusing on the 1614 cm^−1^ peak.

As such, nine 3D printed discs (3 polymer grades: HPC-SSL, HPC-SL, and HPC-L; *n* = 3) were scanned using both NIR and Raman spectroscopy setups. For NIR spectroscopy, the results showed a similar trend in the change in peak morphology and intensity at 5800–5900 cm^−1^, initially indicating its potential suitability for use in the calibration model (Figure 8). As seen previously, the low molecular weight HPC grade (HPC-SSL) exhibited a double peak morphology, suggesting a higher degree is present in the crystalline form, which was in alignment with the previous findings from XRPD (Figure 2). For Raman spectroscopy, the results were consistent with this finding, indicated by the increase in peak intensity at 1614 cm^−1^ for HPC-SSL compared with HPC-L and -SL formulations (Figure 9).

**Figure 10:**
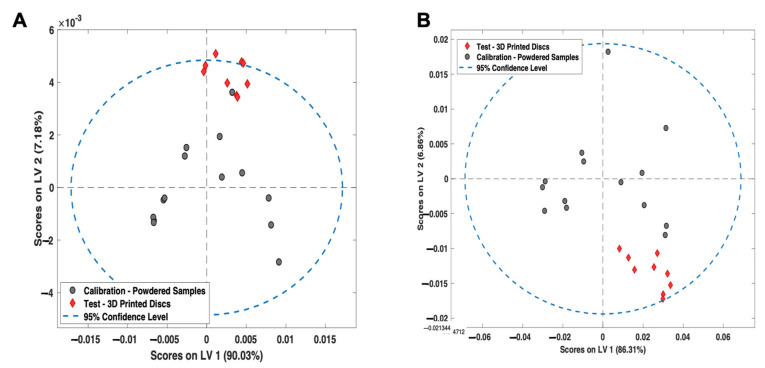
LV1 vs. LV2 Scores plot for the (**A**) NIR spectroscopy PLS model and (**B**) Raman spectroscopy PLS model. Grey points = calibration samples; red points = 3D printed discs.

To evaluate the suitability of both the PLS models for predicting the amorphous content of 3D-printed discs, the models were qualitatively analysed using LV1 vs. LV2 score plots (Figure 10A,B). For Raman spectroscopy, all the 3D printed samples were within the graph 95% confidence interval (CI) for LV1 vs. LV2 score plots. These results suggest that the PLS models are fit-for-purpose for the evaluation of 3D-printed discs. For NIR spectroscopy, two of the samples were found to be marginally outside of the 95% confidence interval, which is likely due to NIR spectroscopy being more sensitive to the physical presentation of the sample compared to Raman spectroscopy.

Test powdered samples containing a known amount of amorphous itraconazole (4%, 8%, 12%, and 16% *w*/*w*), as well as HPC-L, HPC-SL, and HPC-SSL 3D printed discs were scanned and input into the PLS calibration models developed using NIR and Raman spectroscopy (Table 4). The results for the powdered samples indicated a close agreement between the actual and predicted amorphous content values, as well as agreement between the NIR and Raman results (differences of 0.19%, −0.07%, −0.7%, and −0.03% for powders loaded with 4%, 8%, 12%, and 16% *w*/*w* amorphous itraconazole, respectively).

The results for the 3D-printed discs showed a close agreement between the NIR and Raman results (differences of 1.00%, −0.78%, and +0.77% for discs printed with HPC-L, HPC-SL, and HPC-SSL, respectively), with the amount of amorphous conversion occurring as a function of polymer molecular weight: HPC-L > HPC-SL > HPC-SSL, a trait which was observed in the previous qualitative NIR, Raman, and XRPD analyses. However, the error observed between NIR and Raman results for the 3D printed discs was found to be higher than for the powdered samples, which is likely due to the difference in surface presentation between the calibration samples and printed discs. Furthermore, it is worth noting that these results are only indicative, and application in practice would require robust validation by comparing these findings to a reference method (such as quantitative DSC).

## 4. Conclusions

Overall, this paper demonstrates the ability of SLS 3DP to create amorphous solid dispersions of a BCS Class II drug (itraconazole) with three different HPC polymer grades (HPC-L, HPC-SL, and HPC-SSL). Interestingly, the polymers with higher molecular weights (HPC-L and -SL) underwent a uniform sintering process, attributed to the increased particle size, and, hence, had improved flow characteristics compared with the lower molecular weight grade (HPC-SSL), which demonstrated craters and defects on the formulation surface. XRPD analyses found that the SLS 3DP process caused an amorphous conversion of itraconazole with all three polymers, with HPC-SSL retaining a small amount of crystallinity, likely due to the ineffective clearance of unsintered material on the drug product surface. NIR and Raman spectroscopy were explored and identified as promising non-destructive and rapid techniques to measure the amount of amorphous drug conversion, qualitatively and quantitatively, in powdered samples and SLS 3D-printed discs.

## Figures and Tables

**Table 1:** Drug calibration sample compositions.

Sample Code	Crystalline Itraconazole (% *w*/*w*)	Amorphous Itraconazole (% *w*/*w*)	HPC Polymer (% *w*/*w*)	Candurin Gold Sheen (% *w*/*w*)
Cal 1	20	0	77	3
Cal 2	16	4	77	3
Cal 3	12	8	77	3
Cal 4	8	12	77	3
Cal 5	4	16	77	3
Cal 6	0	20	77	3

**Table 2:** Physical properties of the 3D printed dosage forms.

Formulation Code	Weight ± SD (mg)	Breaking Force ± SD (N)	HPLC Recovery ± SD (%)
HPC-L printlets	80.75 ± 0.49	22.0 ± 3.6	99.26 ± 0.04
HPC-SL printlets	88.55 ± 11.95	16.7 ± 2.1	99.67 ± 2.06
HPC-SSL printlets	90.4 ± 6.5	13.3 ± 3.1	100.94 ± 0.88

**Table 3:** Flow properties of pure HPC polymers and formulation blends.

Formulation Code	Bulk Density ± SD (g/mL)	Tapped Density ± SD (g/mL)	Compressibility Index (%) ± SD	Hausner Ratio ± SD	Powder Flow
Itraconazole	0.26 ± 0.01	0.52 ± 0.01	50 ± 0.96	2.00 ± 0.04	Very poor
HPC-L	0.39 ± 0.02	0.51 ± 0.03	23.8 ± 4.74	1.31 ± 0.08	Passable
HPC-SL	0.35 ± 0.00	0.45 ± 0.02	22.7 ± 4.17	1.29 ± 0.07	Passable
HPC-SSL	0.37 ± 0.01	0.52 ± 0.02	28.6 ± 3.61	1.40 ± 0.07	Poor
Itra 20% *w*/*w* HPC-L blend	0.36 ± 0.02	0.47 ± 0.01	24 ± 4.06	1.32 ± 0.07	Passable
Itra 20% *w*/*w* HPC-SL blend	0.39 ± 0.01	0.51 ± 0.02	24 ± 0.32	1.32 ± 0.01	Passable
Itra 20% *w*/*w* HPC-SSL blend	0.32 ± 0.04	0.50 ± 0.04	36 ± 2.60	1.56 ± 0.06	Very poor

**Table 4:** Actual vs. predicated amorphous itraconazole content of test validation powdered samples (*n* = 4) and 3D-printed discs (*n* = 9) predicted using NIR spectroscopy and Raman spectroscopy.

Sample Code	Amorphous Content (% *w*/*w*)		
	Actual	NIR	Raman
Test Validation 1 (powder)	4	3.89	4.08
Test Validation 2 (powder)	8	7.94	7.87
Test Validation 3 (powder)	12	12.23	11.53
Test Validation 4 (powder)	16	15.98	15.95
HPC-L 3D-printed disc	-	18.39 ± 0.90	17.39 ± 0.84
HPC-SL 3D-printed disc	-	17.39 ± 0.56	16.61 ± 0.90
HPC-SSL 3D-printed disc	-	12.39 ± 0.57	12.86 ± 1.12

## Data Availability

Not applicable.
